# Inhibitory control and academic achievement – a study of the relationship between Stroop Effect and university students’ academic performance

**DOI:** 10.1186/s40359-024-01984-3

**Published:** 2024-09-27

**Authors:** Martin Dvorak

**Affiliations:** Södertörn University, Stockholm, Sweden

**Keywords:** Academic performance, Executive functions, Inhibitory control, Stroop Test, Manifested conscientiousness

## Abstract

While previous research has identified executive functions as predictors of academic performance in school children, similar studies conducted among adults show mixed results. One of the reasons given for executive functions having a limited effect on academic achievements in adulthood is that they are usually fully developed by that time. Since these executive functions are at their peak at that age, the individual differences in these as well as their influence on academic performance in adults are harder to trace. The paper describes a study conducted among 107 university students the goal of which was to find out whether there is any relationship between the adult students’ inhibitory control values measured with the Stroop Test and their academic achievements. Although the results indicate a weak correlation between the Stroop Effect and the students’ academic performance of low statistical significance, which seems to confirm the outcomes of the previous studies focusing on adults, the study reveals an unexpected statistically significant correlation between the students’ grade averages and the number of their incorrect color identifications. This phenomenon appears to be worth pursuing in future research since it suggests the existence of another, relatively quickly measurable, variable possibly reflecting other predictors of academic performance in adults such as a degree of their manifested conscientiousness, their ability to concentrate on an assigned, relatively short, one-off task and their attitude to fulfilling this task. The Stroop Test, despite not being originally designed for this purpose, might thus be used as a simple tool suitable for providing information about these variables via the subject’s number of color identification errors. Such information can subsequently inform the activities that educators may include in their curricula to foster conscientiousness and concentration in the students lacking these.

## Introduction

As documented by previous research, academic performance as the “level of knowledge demonstrated in an area or subject compared to the norm for the particular age and level of education” [46] has been affected by a myriad of factors. These are socioeconomic [66], student-related (e.g. students’ self-control and class attendance) [25, 29], or psychosocial [60]. Another factor documented as affecting academic performance is a complex of executive functions which appear to play a significant role in language development as well as the processing and organization of received information [57]. The processing of received information is done either through automatic attention or controlled attention. In the case of the former, the attention responses direct attention automatically to a target regardless of concurrent inputs or memory load. In the case of the latter, an active attention of the subject is required, which also makes the information processing limited in terms of processing capacity [63].

Executive functions encompass cognitive skills related to attention control, i.e. the process by which attention is selectively directed to specific aspects of a representation, particularly in misleading situations [11]. One of the attention control mechanisms can be switching attention between tasks where, in the case of a card-sort test, for instance, the subject must switch between different rules by which they sort cards (e.g. first by their shape and then by their color). Another mechanism is the inhibition of attention when it comes to the stimuli that need to be ignored. This inhibition is activated in, for instance, multilinguals when they need to suppress their temptation to use one (or more) of their languages not needed or inappropriate for a given situation [11, 13, 77, etc.]. Other researchers [2, 53, etc.] work with the terms of inhibition (the ability to suppress dominant responses, a term synonymous to the attention inhibition mentioned above), shifting (the ability to switch over between tasks, a term synonymous to switching attention mentioned above) and monitoring (the ability to update information in the working memory). The working memory as a function that enables individuals to temporarily remember information while competitively processing information [54] has been mentioned as a factor influencing school performance even more than intelligence with the latter predicting a wide range of indicators of academic success [48, 52].

### Effect of executive functions on academic performance

Multiple studies emphasize the fact that educational research should pay more attention to executive functions since these represent essential ingredients for successful academic functioning [75] and since they also appear to be connected with school dysfunction as deficits in them have been associated with disabilities in mathematics and reading [51, 70]. According to Pascual [57], who also mentions cognitive flexibility, i.e. the ability to temporarily manipulate information, and planning, the executive functions represent “distinct, but related, higher-order neurocognitive processes that control thought and behaviors aimed at achieving an objective goal” (p. 2). The existence of relationship between executive functions and academic achievement is also supported by other studies most of which investigate this relationship in children of pre-school or early-school age (e.g. [4, 9, 19, 28, 76, etc.]) or those that study it in the context of learning disabilities [3, 37, 49, 64].

Some studies also stress the fact that the positive contribution of executive functions to academic performance is domain-dependent, i.e. that certain executive functions contribute to gaining certain knowledge or skill more than others. Thus, inhibition, for instance, appears to be beneficial when it comes to mathematics and science [57]. Similarly, Gerst [38] found a direct relationship between inhibition and the ability to conduct mathematical calculations in children aged 10–11.

As there seems to be a variation in the way in which younger and older children solve calculations due to the age-related shift from the procedural-based processing in arithmetic tasks to more memory-based [72, 7, 16, etc.] it is also different types of interference that appear to disrupt children at different ages. In a dual-task study McKenzie et al. [50], for instance, found out that the mathematical processing of 6-year-old children was disrupted only by a visuo-spatial passive interference task whereas in the 8-year-old ones it was disrupted by both a visuo-spatial and a phonological interference task. In this respect, the type of information processing deployed in problem solving appears to determine the type of irrelevant stimuli that need to be suppressed through inhibition for the students to complete an arithmetic task efficiently.

The executive function of inhibition is usually defined as the ability to suppress dominant but irrelevant responses and prioritize important information instead. This way “it moderates behavior, suppresses impulsive reactions to a stimulus, and enables an appropriate and thoughtful response” [57]. Cognitive inhibition is thus responsible for planning, analyzing and choosing the most appropriate response.

### Negative effects of a low degree of inhibitory control on academic performance

The relationship between poor academic performance and poor performance in tasks requiring the inhibition of irrelevant information has been pointed out by multiple studies. Espy et al. [33], investigating how working memory and inhibitory control affect arithmetic competency, identified differences in the ability to inhibit irrelevant stimuli as a factor responsible for unique variance in mathematical skills. In mathematics, inhibitory control is used to inhibit information that should not be maintained in working memory for upcoming responding [27]. Similarly, Espy et al. point out that to flexibly shift responding in the face of conflicting rules requires maintaining the rule in mind and inhibiting prepotent, previous responses [33]. Passolunghi & Lanfranchi [55] mention inhibition as a factor influencing performance at the numerical competence test.

The importance of the role that inhibition plays in reading and listening comprehension has been pointed out by studies focusing on children. Passolunghi et al. [56], for instance, stress that groups of poor problem solvers tend to perform poorly in a working memory test requiring inhibition of irrelevant information and that this condition appears to be related to poor recall of critical information and greater recall of to-be-inhibited information. In addition, the process of reading often involves exposure to visual distractions such as images, graphs, etc. present in the very text as well as external physical distractors in the environment in which the reading takes place. In such situations, the inhibitory control helps the reader to stay focused on the written content. Similarly, De Beni et al. [26], showed that the “poor comprehenders” had a significantly lower performance in the listening span test associated with a higher number of intrusions. These intrusions can be background noise or competing sounds that need to be ignored for the listener to focus on and understand what a speaker is saying. In addition, both reading and listening often involve interpretation of figurative language, where the inhibition of the literal, irrelevant information enables the reader or listener to grasp the relevant meaning [39].

### Inhibitory control as a facilitator of learning

The positive correlation between a degree of inhibitory control and academic achievement has been documented by other studies as well. Duckworth et al. [29], for instance, stress behavioral inhibition (self-control) as affecting academic performance. St Clair-Thompson & Gathercole [62] identify inhibition as a factor associated with achievement in English, mathematics, and science in 11- and 12-year-old children while Blair & Razza [14] point out that the inhibitory control correlated with both early math and reading ability in their study conducted among 3- to 5-year-old children. Privitera et al. [59] give a reason for why the improved inhibitory control leads to greater academic performance; the students with improved inhibitory control can focus on tasks both within and outside of the classroom better, ignoring the ever-growing number of distractions present in their environments. The authors also claim that this improved focus may result in superior academic performance. Irvan & Tsapali [44] point out the positive effect of improved inhibitory control on academic performance stating that the inhibition as an executive function appears particularly crucial for young children growing up and learning as they are exposed to constant distractions vying for their attention. Blair & Razza [14] also suggest that curricula designed to improve self-regulation skills and enhance early academic abilities may be most effective in helping children succeed in school.

### Age-dependent effect of executive functions on academic performance

On the other hand, some sources conclude that the relationship between executive functions, with inhibitory control representing one of them, and academic performance appears to depend on age. Bryce et al. [15] focused on the relationship between executive functions and metacognitive skills, which they have identified as most significant predictors of educational achievements in their study groups of 5- and 7-year-old children. Their results indicate that executive functions appear to be more related to metacognitive skills in 5-year-olds than in 7-year-olds. In the study conducted among subjects aged 5–17, Best et al. [10] analyzed a varying correlation between executive functions and academic achievement in relation to age concluding that the correlation is strongest at the ages of 6, 8–9 and subsequently appears to be of somewhat consistent strength in the late childhood and adolescence. This conclusion partly contradicts the findings of Altemeier et al. [1], who claim that the effect of executive functions on academic performance may be more evident earlier in schooling, when academic skills are less automatic and require more effortful planning to execute. Similarly, some other studies point out inhibition as the strongest predictor of academic success at children’s early age such as that by Senn et al. [61]. They found out that while working memory contributed to academic success to a greater extent in older children, the inhibitory control did this in younger ones. Other authors [8, 42, etc.] mention the complete maturation of inhibitory processes by around the age of 12. The decrease in the potential of executive functions to predict academic performance during secondary education and even more so during university studies has also been touched upon by Pascual & Robres [57].

### Methods used to measure the inhibitory control and the role of anterior cingulate

Scientists have developed several methods to measure inhibitory control, whose choice partly depends on the type of inhibition that is being targeted. Response inhibition, a term referring to the process of countermanding a prepotent motor response, has generally been assessed using non-selective stopping tasks such as the stop signal, go/no-go, and anti-saccade tasks. These tests require participants to intermittently suppress a motor response to a given presentation of a conditional stimulus or cue [6, 20, 71, 73]. Attentional inhibition, which refers to the ability to resist interference from stimuli in the external environment, has been investigated using visual matching tasks requiring participants to judge whether target and comparison stimuli are the same or different and, at the same time, requiring them to ignore task-irrelevant distracters [36, 68, 71].

Response inhibition and attentional inhibition are also commonly measured with Stimulus-Response Compatibility tasks, such as the Eriksen Flanker (Flanker), Simon, and Stroop tasks [32, 65, 69, 71]. The Stroop task (test) is utilized for comparing reaction times to stimuli in the condition where this control is not deployed (congruent condition) with the reaction times requiring inhibiting irrelevant stimuli (incongruent condition). The test has been shown to activate either the left dorsolateral prefrontal cortex or anterior cingulate for cognitive inhibition [74]. As Imbrosciano & Berlach [43] point out, anterior cingulate is considered to be responsible for selecting an appropriate response when the brain is exposed to two conflicting conditions. Bush et al. [17] hypothesize that anterior cingulate dysfunction is responsible for producing core features of ADHD, namely inattention and impulsivity. Anterior cingulate activation has been linked to detection of conflict and its resolution [18] as well as to academic results in college students [40]. The last-named researchers investigated the activity of anterior cingulate in connection with the error-related negativity (ERN, an electrophysiological signal associated with the anterior cingulate monitoring process, occurring approximately 100 ms after an error is made) and found a correlation between the magnitude of ERN and undergraduate students’ academic performance suggesting that the error detection mechanism is stronger in the students who perform better at university. Veroude et al. [74] observed a positive correlation between average course grades and the activation of anterior cingulate cortex in freshmen enrolled in a medical college during cognitive inhibition on the Stroop task finding no relationship between the course grades and activation of the left dorsolateral prefrontal cortex. Similarly, there are other studies which suggest a link between inhibitory control and academic performance associating the activation of anterior cingulate with cognitive control across tasks (e.g [31, 34]).

### The aim of the study

The aim of the study described in this paper was to find out whether there is any relationship between *adult* students’ inhibitory control measured with the Stroop Test and their academic achievement. To measure the degree of inhibitory control, a computerized version of the Stroop Test was used.

Based on the previous research (e.g. [29, 40, 55, 57, 74] the initial hypothesis was that there might be a relationship between inhibitory control and academic performance. In this respect, the participants with a higher degree of inhibitory control (lower Stroop Effect) were expected to be those with a higher grade average and lower failure rate than those indicating a lower degree of inhibitory control (higher Stroop Effect). On the other hand, if the correlation between the two variables were to be found, it was not expected to be significantly high since the previous research studying this phenomenon in relation to age points to the inhibitory control exerting its influence on academic performance chiefly at an individual’s young age [1, 4, 9, 19, 28, 76].

## Method

### Participants

107 students studying at (undisclosed) University, Stockholm, Sweden, (29 males, 78 females, mean age = 25.83 years, SD age = 6.32 years, age range = 19–52 years) participated in the study. Originally, 110 students were involved in the study, but 3 of them were removed as outliers due to the overwhelming majority of their grades being at the extreme ends of the grading scale, i.e. either VGs or Us, and only few Gs (for more information on the grading scale, see the next section). This was done to exclude the students whose extraordinary performance in certain academic subjects might be due to either their extra talents for, or their exceptional motivation to study, these subjects. The participants were recruited from students each of whom was enrolled in one of three teacher education programs, i.e. either primary (*N* = 21), secondary (*N* = 51), or upper secondary (*N* = 35). The reason why the participants were recruited from this group was that most of the courses they study within these programs are somewhat similar in terms of contents. Besides, the students are also assessed in these courses mainly by the same teachers. The original idea was to recruit the highest number of volunteers enrolled in the three programs who were, at the same time, studying the courses given by the department in which the study was conducted. Nevertheless, the final number of the participants was determined by their willingness to participate in the study and it was also restricted by the fact that all of them had to be tested on campus in a computer laboratory within the limited time of the project. The volunteers had no neurological or psychiatric disorders. All the participants signed an informed consent with their participation in the study.

### Data collection and analysis

Information about the participants’ age and sex was collected via questionnaires distributed among the participants prior to the execution of the Stroop Test. The students’ university grade averages were computed based on their past course grades and their calculation included a computational model (see below) used in another study [30] researching the effect of mother tongue proficiency on the students’ academic performance.

The study was conducted in an institution using the grading scale consisting of three grades, i.e. VG, G, and U, a system commonly used in Swedish universities. According to this system, VG represents “passed with distinction,” G denotes “passed,” and U denotes “failed.” To facilitate a statistical analysis, these grades were assigned numerical values of 4, 2, and 0, respectively. This approach mirrors the GPA calculation method, where the highest grade corresponds to 4, the middle one to 2, and the fail grade to 0. Each student’s failure rate, expressed as a percentage, was computed as the ratio of their fail grades (Us) to the total number of grades received. Approximately 30 grades, encompassing both courses and graded modules, were considered for the computation of grade averages and failure rates per student. In instances where a student received multiple fail grades for the same course or module, each of these was included as a distinct grade in the calculation.

To measure the participants’ inhibitory control, a computerized version of the Stroop Test available at https://www.psytoolkit.org/ was used. The task was performed in English since the students represent a relatively uniform group when it comes to their English knowledge, which is at the C1 level of Common European Framework of Reference for languages. Moreover, English represents the language that all the participants have studied in a language instructional setting and thus the color identification rule in the Stroop test had to be followed in the context of their knowledge previously adopted at school. In this respect, the experiment made the participants deploy controlled information processing [63] through the application of a new cognitive concept requiring the inhibition of the semantic contents they have learnt at school before. This way an attempt was made at inducing the situation activating those cognitive processes that resemble the ones which are in operation in school environments when new concepts are learnt or when adjustments are made to the already acquired knowledge.

The task consisted of two conditions on which the participants were tested: (a) congruent trials, where the names of colors displayed on the screen matched the colors these were displayed in, (b) incongruent trails, where the names of colors displayed on the screen did not match the colors these were displayed in. For each trial type the students were instructed to identify the color of the word as quickly as possible by pressing a corresponding key on their keyboards. The keys the subjects were instructed to press were those that bore the initial letters of the names of the colors the words were displayed in. Therefore, when the word “red”, for instance, got displayed in blue, the students were supposed to press the *b* key (“b” standing for “blue”). The explicit instruction given to the students was to disregard the meaning of the words and focus solely on the color in which these words were displayed.

There were four colors used in the test (red, yellow, blue and green) and the students were instructed to press the *r*,* y*,* b* and *g* keys, respectively, to indicate these. Before each of the words was presented in the middle of the screen against the black background (for up to 2 s or until the participant responded), a fixation cross was displayed in the same position for 200 milliseconds for the participant to know where the word would appear. Once the participant made their choice, either a word “correct” or “wrong” popped up for 500 milliseconds depending on whether their choice had been correct or not. The computer script in which the test was run measured the participants’ reaction times in both the congruent and incongruent conditions and counted the errors they made when indicating a wrong color. The Stroop test was run under these conditions twice – once as a practice session with thirty trials, whose purpose was to make sure all the participants understood what they were supposed to do as well as to enable them to practice the key-color associations, and then as the test itself with 120 test trials. Half of the test trials were in the congruent condition and the other half in the incongruent one. The congruent and incongruent conditions were mixed and presented to the subjects randomly.

The main Stroop effect size was calculated for the individual participants according to the following formula using the reaction times recorded for congruent as well as incongruent trials:

$$\eqalign{{\rm{Stroop}}\,{\rm{Effect}}\,{\rm{ = }} & {\rm{Incongruent}}\,{\rm{Trials}}\,{\rm{Total}}\,{\rm{Time}}\,{\rm{-}} & {\rm{Congruent}}\,{\rm{Trials}}\,{\rm{Total}}\,{\rm{Time}} \cr}$$

The reaction times used in the formula above were collected together with the numbers of incorrect color identifications from files saved on a server once the tests had been completed.

Subsequently, Pearson’s bivariate correlation analysis was conducted on the collected data to find out the correlation coefficients between the participants’ grade averages (as well as failure rates) and their Stroop Effect values. Similar analyses were also done for their reaction times in congruent and incongruent conditions as well as their number of Stroop Test mistakes, i.e. the situations where a color was not correctly identified.

As the study group the analysis was conducted with consisted of three sub-groups of students (each sub-group consisting of students enrolled in one of the three teacher training programs), One-Way ANOVA was used to compare these sub-groups for Stroop Effect, reaction times for congruent trials, reaction times for incongruent trials, and number of color identification errors. Since the sub-groups were of unequal sizes, the test of homogeneity of variances was run on all the variables with the subsequent Tukey HSD post hoc test to identify the significance of the differences between the sub-groups.

## Results

The students’ (*N* = 107) mean Stroop Effect, mean reaction times (in ms) in congruent and incongruent conditions, the mean number of errors as well as the mean number of times the participants exceeded the time limit are given in Table 1 below. The table also lists students’ grade average and fail rate average as well as standard deviations for each variable.

**Table 1:** Measured variables

*N* = 107	Mean	SD
Grade Average	2.16	0.70
Fail Rate (in %)	16.33	18.01
Stroop Effect (ms)	130.44	82.18
Congruent condition (ms)	803.34	172.68
Incongruent condition (ms)	933.74	197.73
Error rate (number of times)	7.64	7.82
Time limit exceeded	2.47	4.23

**Fig. 1:**
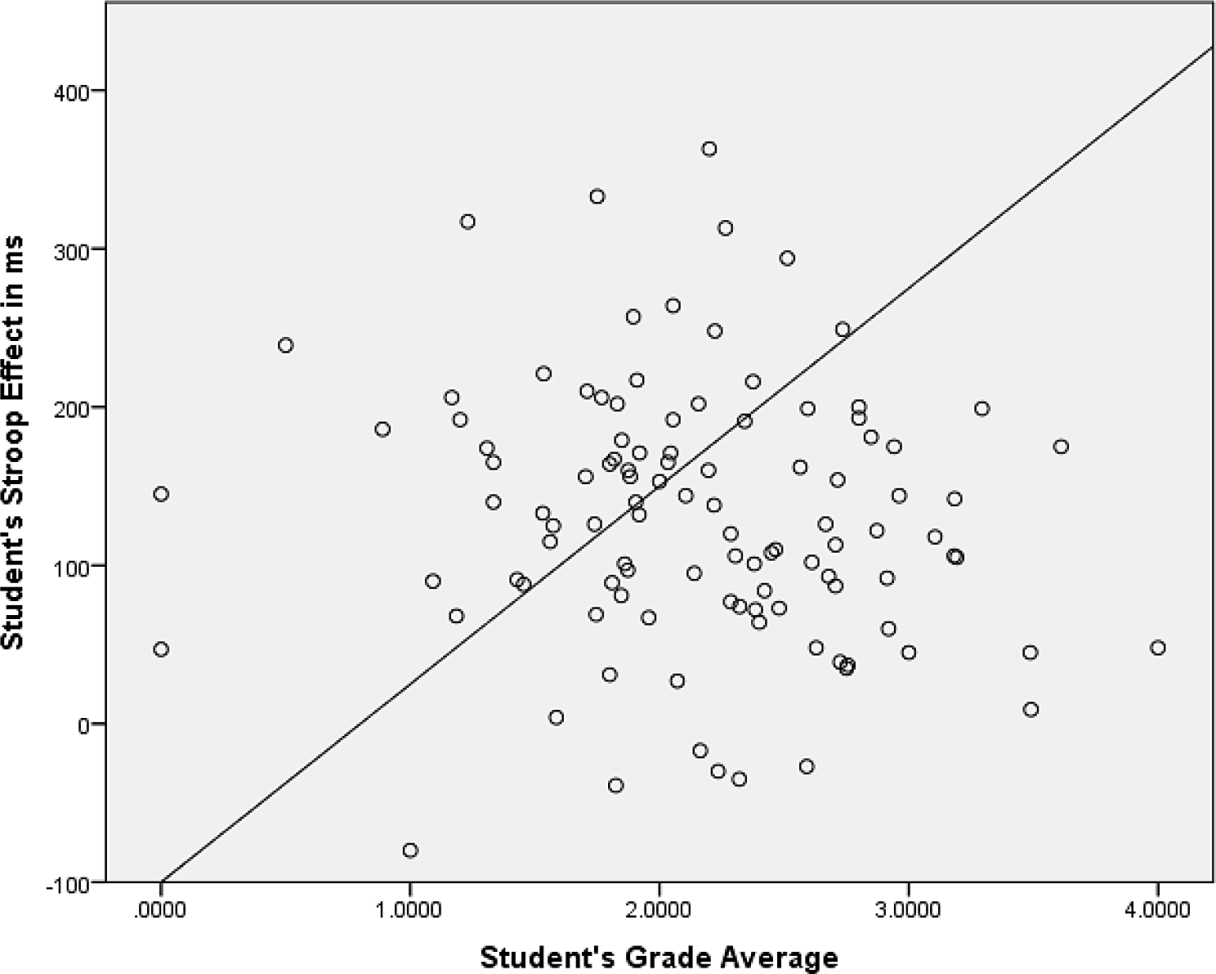
Grade average – Stroop Effect scatter plot

**Fig. 2:**
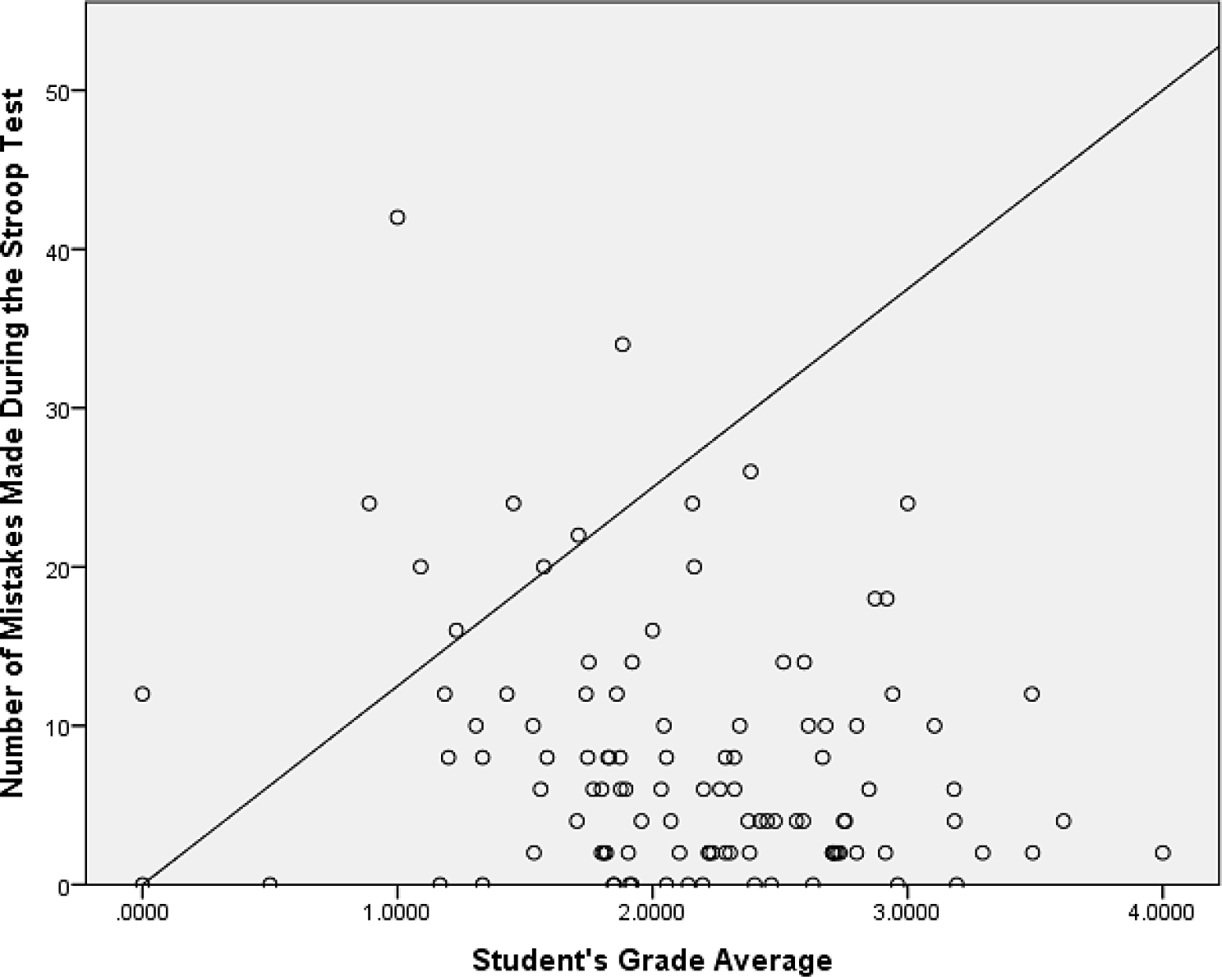
Grade average – Stroop Test errors scatter plot

Pearson’s bivariate correlation analysis shows a very weak negative correlation of low statistical significance, *r*(107) = − 0.13, *p* = .20, between the Stroop Effect and participants’ grade averages and basically no correlation between the former and participants’ fail rate, *r*(107) = 0.04, *p* = .68, (see Table 2 below). Similarly, there seems to be no statistically significant relationship between the participants’ grade averages and their reaction times in congruent (*r*(107) = − 0.06, *p* = .58) and incongruent (*r*(107) = − 0.10, *p* = .31) conditions as well as no significant relationship between the grade averages and the number of times the participants exceeded the time limit for color identification (*r*(107) = − 0.07, *p* = .46). The only statistically significant relationship detected appears to be the one between the students’ grade averages and the number of mistakes (incorrect color identifications) made during the Stroop Test with a weak negative correlation, *r*(107) = − 0.21, *p* = .03. Scatter plots for Stroop Effect values and the number of errors made during the test in relation to the students’ grades are shown in Figs. 1 and 2 below. The diagonal lines in the scatter plots represent identity lines indicating the points in which the values of the variables correlate perfectly while the distances of the data points from these lines represent degrees of correlation; the closer the points are to the line, the stronger the correlation between the variables. Tables 3 and 4 show the comparison of measured variables across the educational programs and related post hoc Tukey HSD, respectively.

**Table 2:** Grade average correlations

*N* = 107	Correlation	Significance
Grade Average – Stroop Effect	*r*(107) = − 0.13	*p* = 0.20
Fail Rate – Stroop Effect	*r*(107) = 0.04	*p* = 0.68
Grade Average – Congruent Trials	*r*(107) = − 0.06	*p* = 0.58
Grade Average – Incongruent Trials	*r*(107) = − 0.10	*p* = 0.31
Grade Average – Stroop Test Errors	*r*(107) = − 0.21	*p* = 0.03*
Grade Average – Time Limit Exceeded	*r*(107) = − 0.07	*p* = 0.46

* Significant correlation

**Table 3:** Stroop effect, time for congruent trials, time for incongruent trials, and color identification errors – comparison across educational programs

The program the students are enrolled in		Stroop Effect in ms	Reaction Time for Congruent Trials	Reaction Time for Incongruent Trials	Number of Color Identification Errors
Primary	Mean	174.76	801.90	976.62	2.52
	N	21	21	21	21
	SD	84.29	180.97	227.46	2.79
Secondary	Mean	133.00	823.69	956.63	4.49
	N	51	51	51	51
	SD	83.95	164.03	172.98	3.59
Upper-Secondary	Mean	100.11	774.54	874.66	3.63
	N	35	35	35	35
	SD	65.90	180.72	204.80	4.74
Total	Mean	130.44	803.34	933.74	3.82
	N	107	107	107	107
	SD	82.18	172.68	197.73	3.91

The Tukey HSD post hoc test (see Table 4 below) shows the comparison of the distribution of the values within the sub-groups, where the only statistically significant Stroop Effect mean difference of 74.65 ms (*p* = .002) can be found between the primary and upper-secondary programs (the students of the latter indicating a lower mean).

**Table 4:** Stroop effect, time for congruent trials, time for incongruent trials, and color identification errors – post hoc Tukey HSD

Dependent Variable	Sub-Group (Program)	Sub-Group (Program)	Mean Difference	Std. Error	Sig.
Stroop Effect in ms	Primary	Secondary	41.76	20.37	0.106
		Upper-Secondary	74.65^*^	21.69	0.002
	Secondary	Primary	-41.76	20.37	0.106
		Upper-Secondary	32.89	17.24	0.142
	Upper-Secondary	Primary	-74.65^*^	21.69	0.002
		Secondary	-32.89	17.25	0.142
Reaction Time for Congruent Trials	Primary	Secondary	-21.78	44.84	0.878
		Upper-Secondary	27.36	47.74	0.835
	Secondary	Primary	21.78	44.84	0.878
		Upper-Secondary	49.14	37.96	0.401
	Upper-Secondary	Primary	-27.36	47.74	0.835
		Secondary	-49.14	37.96	0.401
Reaction Time for Incongruent Trials	Primary	Secondary	19.99	50.58	0.918
		Upper-Secondary	101.96	53.84	0.146
	Secondary	Primary	-19.99	50.58	0.918
		Upper-Secondary	81.97	42.82	0.140
	Upper-Secondary	Primary	-101.96	53.84	0.146
		Secondary	-81.97	42.82	0.140
Color Identification Errors	Primary	Secondary	-1.97	1.00	0.128
		Upper-Secondary	-1.10	1.07	0.558
	Secondary	Primary	1.97	1.00	0.128
		Upper-Secondary	0.862	0.85	0.570
	Upper-Secondary	Primary	1.10	1.07	0.558
		Secondary	− 0.86	0.85	0.570

* The mean difference is significant at the 0.05 level

## Discussion

The goal of this study was to find out whether there was some relationship between the inhibitory control of the students studying at (undisclosed) University, Stockholm, Sweden, measured with the Stroop Test and their academic performance. Based on the previous research focusing on links between school performance and executive functions [51, 70, 75], the hypothesis was that the participants with a higher grade average and lower fail rate might tend to manifest a higher degree of inhibitory control indicated with a lower Stroop Effect. Nevertheless, this correlation was expected to be weak in the study group consisting of university students since the other research into the area shows the strongest correlation between the inhibitory control and the academic performance in subjects at their early age [8, 10, 15, 42, 61].

The results show that although there is a very weak negative correlation (*r*(107) = − 0.13) between the Stroop Effect and participants’ grade averages, which might suggest some effect of the degree of their inhibitory control on their school performance that is in line with the previous research focusing on this phenomenon, this relationship has not turned out to be statistically significant (*p* = .20). As regards the students’ failure rates, these turned out to be completely independent of the Stroop Effect values.

As regards the comparison of the distribution of all the observed values within the different sub-groups of students depending on what program they study, the only statistically significant difference was found in Stroop Effect (mean difference of 74.65 ms (*p* = .002)) between the primary and upper-secondary programs, with the latter indicating a lower Stroop effect.

Another statistically significant relationship detected was the one between the students’ grade averages and the number of mistakes (incorrect color identifications) made during the Stroop Test with a weak negative correlation (*r*(107) = − 0.21, *p* = .03) indicating that the students performing worse academically (having lower grades) made more mistakes during the test. This relationship may suggest that the degree of conscientiousness the students approach the assigned task of Stroop Test with might be in direct proportion to the degree of conscientiousness they approach their university studies with in general. That is [41], point out that conscientiousness predicts better performance on the Stroop task in terms of fewer errors and diminished incongruency effects. They even suggest that this personality trait may promote certain attentional processes even as cognitive capacities decline at a later age. Similarly, other studies [45, 67], deploying other attention control tasks, also found a relationship between conscientiousness and cognitive performance. Finally, as the myriad of studies shows conscientiousness, defined as dependability and will to achieve, as being in direct relationship with academic performance as well [21, 23, 24, 58, etc.], the results of the study described in this paper might be indicative of the newly found relationship between the number of color identification errors (as a factor reflecting this conscientiousness) and academic performance, albeit this relationship has not been studied before.

One of the reasons why the weak correlation between the students’ Stroop Effect and their school performance shows low statistical significance may be that the grade averages had been calculated from the grades the students obtained for a wide range of school subjects ranging from mathematics, to languages, history, etc. That is, the meta-analysis conducted by Pascual & Robres [57] shows that the degree to which executive functions affect school performance depends on the subject studied. This phenomenon can be observed, for example, in the relationship between mathematics and the visuo-spatial aspect of working memory. Similar observations have also been made when it comes to other executive functions, which appear to be more related to performance in mathematics than in a language, for instance. Moreover, the meta-analysis points out that most studies identify working memory as a better predictor of school performance than inhibition and that executive functions represent an important predictor of academic performance and future learning problems at an early age. However, the predictive capacity of executive functions in relation to academic performance seems to decrease during secondary education and even more so during university education, which might be the case with the study described in this article. The reason for this phenomenon could be minimal individual differences in executive functioning in certain age groups. Bialystok [12], for instance, in her study of the Stroop task performance, mentions no differences in Stroop Effect among university undergraduates giving the cognitive performance in this age group being at its peak as a reason for the phenomenon. Likewise, Comalli et al. [22] demonstrated that older adults and children indicate longer response latencies than young adults. The aforementioned factors are also suspected of being the reasons why no correlation has been found between the students’ Stroop Effect and their failure rates.

The fact that the relationship between the students’ Stroop Effect and school performance shows low statistical significance might also be due to the Stroop Test activating different regions of the brain in different individuals – operations that have been documented as correlating with school grades. That is, Veroude et al. [74] report a significant main effect of cognitive inhibition being observed in the left dorsolateral prefrontal cortex, but not so much in the dorsal anterior cingulate cortex (ACC). However, they report the activation of ACC for the “incongruent” condition being associated with higher grades. In this respect, they found an association with achievement only in the situations where the Stroop Test activated dorsal ACC, indicating that “involvement of this region can potentially predict differences in education success.” (p. 104). Other studies also show the involvement of both the ACC and dorsolateral prefrontal cortex during the Stroop task (e.g [47]). even though the ACC does not seem to be necessary for cognitive control as patients with damage to this region perform normally on the Stroop Test [35]. As the study described in this paper did not include functional magnetic resonance imaging, it was not possible to find out in which situations the inhibitory control in the subjects in incongruent conditions resulted from the activation of the ACC and in which situations it resulted from the activation of the dorsolateral prefrontal cortex. Hence, it is also impossible to assess the extent to which the activation of the former or the latter for inhibitory control might influence the subjects’ grades. Not distinguishing between these two conditions could thus have been one of the reasons for the weak p-value of the results and thus it might be desirable to differentiate between them in future research.

Finally, as the current study suggests a link between the number of mistakes made during the Stroop Test and the students’ grade averages, the potential of the test to be used to measure the degree of their manifested conscientiousness and the ability to concentrate on an assigned, relatively short, one-off task should be studied further. The results of such further testing might provide clues regarding to what extent these characteristics can be viewed as predictors of academic performance.

## Conclusion

Overall, this study has shown that there was a weak correlation of low statistical significance between the participants’ grade averages and the inhibitory control measured with the Stroop Test. It has also shown no relationship between their failure rates and inhibitory control.

These findings suggest that differences in the impact of inhibitory control on cognitive functioning among young adults might be much smaller, if any, than in children or older people. This fact seems to be in line with the findings of previous studies which point out that individual differences in executive functions are greatest while these functions are either under development, i.e. in children, or when they are in decline, i.e. in the elderly.

The study has also revealed that the students with lower grades made more color identification errors than those with higher grades. This phenomenon is worth pursuing in the future since the Stroop Test, or any other test where subjects need to follow a relatively simple rule, might be indicative (via their error rates) of their conscientiousness, a way in which they approach a certain assigned task or a degree of their ability to handle the task. Consequently, these findings can offer educators insights into their students’ specific weaknesses in these domains, empowering them to address these areas through tailored teaching approaches, such as individualized activities.

## Data Availability

The data pertinent to this study and used in the analysis are enclosed in a separate file uploaded at the submission of the paper.
